# Supplementary material for: Associations between frontal lobe structure, parent-reported obstructive sleep disordered breathing and childhood behavior in the ABCD dataset
Source: Nat Commun. 2021 Apr 13;12:2205. doi: 10.1038/s41467-021-22534-0 (PMC8044120; doi:10.1038/s41467-021-22534-0)
Supplement: Supplementary file 1 — Supplementary Information [file 41467_2021_22534_MOESM1_ESM.pdf]

Associations between frontal lobe structure, obstructive sleep disordered breathing and childhood behaviour in the ABCD dataset

Amal Isaiah[1,2*], Thomas Ernst[3,4,5], Christine C. Cloak[3], Duncan B. Clark[6] and Linda Chang[3,4,5,7]

[1]Department of Otorhinolaryngology—Head and Neck Surgery, University of Maryland School of Medicine, Baltimore, MD

[2]Department of Pediatrics, University of Maryland School of Medicine, Baltimore, MD

[3]Department of Diagnostic Radiology and Nuclear Medicine, University of Maryland School of Medicine, Baltimore, MD

[4]Department of Medicine, John A. Burns School of Medicine, University of Hawaii at Manoa, Honolulu, HI

[5]Department of Neurology, Johns Hopkins University School of Medicine, Baltimore, MD

[6]Department of Psychiatry, University of Pittsburgh School of Medicine, Pittsburgh, PA

[7]Department of Neurology, University of Maryland School of Medicine, Baltimore, MD

**Corresponding author; email: aisaiah@som.umaryland.edu*

Supplementary Figures 3
Supplementary Figure 1 Relationship between obstructive sleep disordered breathing (oSDB) score and individual Child Behavior Checklist scales (CBCL) in the Adolescent Brain and Cognitive Development Study. 3
Supplementary Figure 2 Mediation effects for the relationship between the frequency of snoring and Child Behavior Checklist (CBCL) measures attributed to regional cortical volumes. 4
Supplementary Figure 3 Frontal lobe regions mediate the relationship between obstructive sleep disordered breathing (oSDB) and abnormal Child Behavioral Checklist (CBCL) outcomes. 5
Supplementary Figure 4 Mediation effects for the relationship between the frequency of snoring and abnormal Child Behavior Checklist (CBCL) measures. 7
Supplementary Tables 8
Supplementary Table 1 Child Behavior Checklist (CBCL) outcomes in the Adolescent Brain and Cognitive Development (ABCD) cohort described by each category subscale. 8
Supplementary Table 2 Effect size estimates for the relationship between baseline covariates and the parent-reported symptoms of obstructive sleep disordered breathing (oSDB). 9
Supplementary Table 3 Effect size estimates for the relationship between obstructive sleep disordered breathing (oSDB) factor score and the volumes of key non-cortical structures. 10
Supplementary Methods 11
Baseline demographics 11
Magnetic Resonance Imaging 11
Use of Generalized Additive Models 12
Mediation Analysis 12
Supplementary References 14

Supplementary Figures

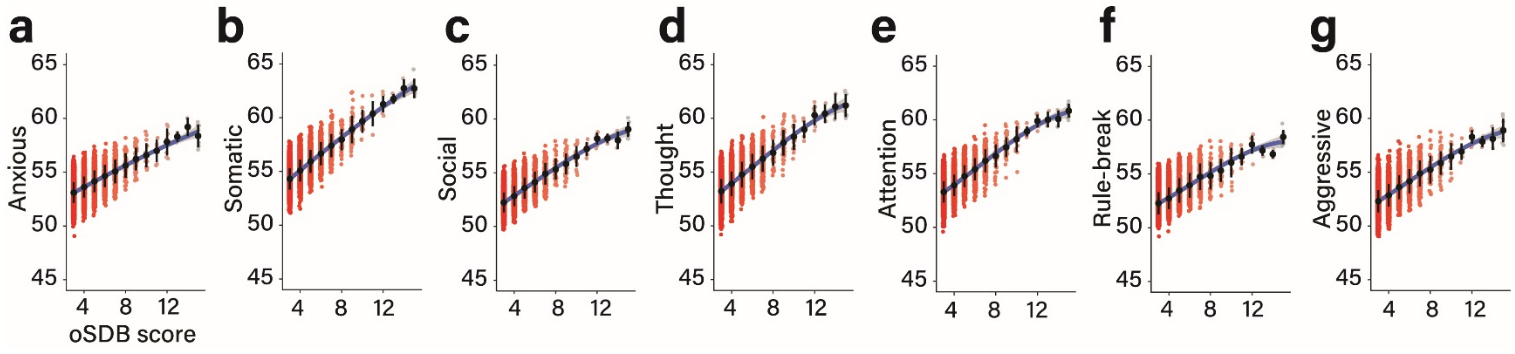

Supplementary Figure 1 Relationship between obstructive sleep disordered breathing (oSDB) score and individual Child Behavior Checklist scales (CBCL) in the Adolescent Brain and Cognitive Development Study. The outcome representing the overall burden of oSDB symptoms was derived by adding the individual symptom frequencies of nighttime breathing problems, gasping and snoring from the Sleep Disturbance Scale for Children. **(a-g)** show the relationships between the oSDB factor score and CBCL syndrome scores. All CBCL measures are adjusted for age, sex, race/ethnicity, the presence of asthma, total household income before taxes as fixed effects and site as a random effect. In each panel**,** the predicted marginal means at each level are fitted with a smoothing spline, with the error bars spanning one standard deviation at each level of the severity of oSDB. The estimates of effect sizes are provided in Figure 1. Source data are provided as a source data file.

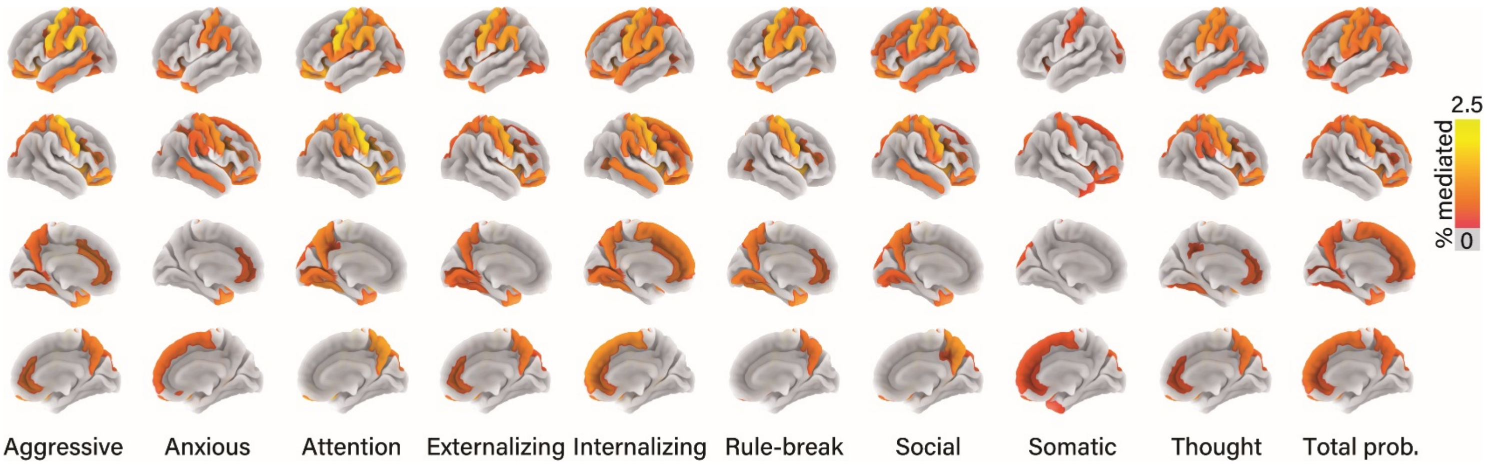

Supplementary Figure 2 Mediation effects for the relationship between the frequency of snoring and Child Behavior Checklist (CBCL) measures attributed to regional cortical volumes. Covariate-adjusted effect size maps show the regional mediated effects that define the association between the frequency of snoring reported on a five-point scale as 1=never, 2=occasional (once or twice a month), 3=sometimes (once or twice a week), 4=habitual (more than twice a week but not daily), and 5=daily. The CBCL scores include the composite and individual syndrome scales as annotated below each panel. The average mediated effects, expressed as a percentage of the total effect, were calculated from bootstrapped confidence intervals derived from joint modeling of two regressions. The first measures the relationship between the frequency of snoring and the morphometric cortical region of interest (ROI), and the second between the ROI variable and the CBCL score. The average mediated effects were projected on four views of atlas-based maps following application of a P value threshold of 0.05 corrected for false discovery. Source data are provided as a source data file.

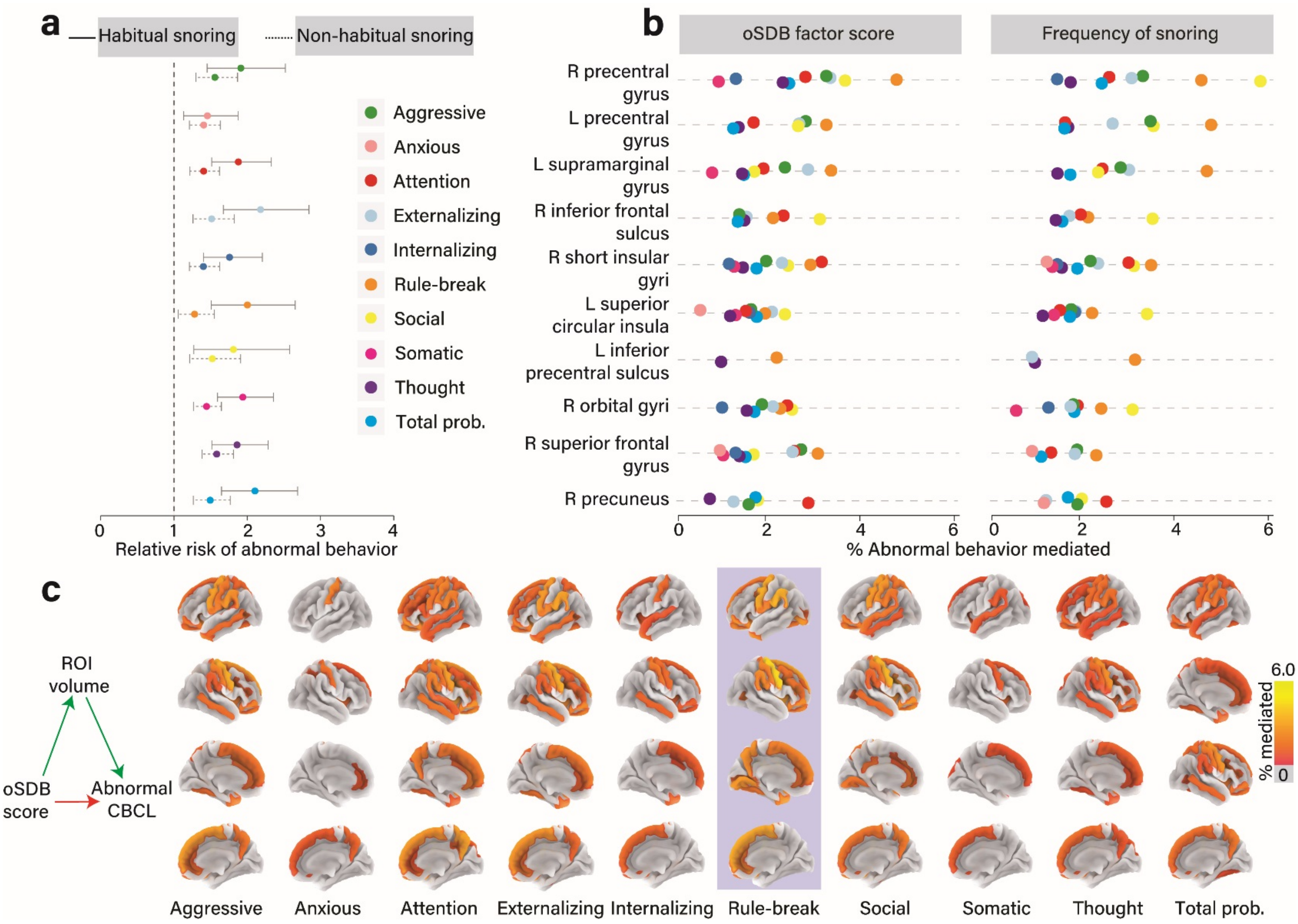

Supplementary Figure 3 Frontal lobe regions mediate the relationship between obstructive sleep disordered breathing (oSDB) and abnormal Child Behavioral Checklist (CBCL) outcomes. (a) shows the point estimates and 95% confidence intervals for the risk of abnormal CBCL score defined by established cut-offs. Habitually snoring children (snoring at least three nights a week) were more likely to have abnormal CBCL values. We then used a mediation model to estimate the extent of the covariate-adjusted relationship between oSDB factor score and abnormal CBCL outcomes (Table S1) apportioned to alterations in cortical volume. These included children who met the cut-off for clinical evaluation with at least a

borderline score. For social, thought, attention and total problems, arbitrary cut-offs of 65 were used. All mediation models included age, sex, race/ethnicity, history of asthma and the total household income before taxes as fixed effects, and the recruitment site and the scanner serial number as random effects. **(b)** shows the top ten mediated effects for cortical regions of interest (ROI) for both oSDB factor score and the frequency of snoring showing similar effects. Most of the mediated effects, especially within the precentral gyri, were observed for ROI volumes and not for thickness or area (not shown). These mediation effects expressed as a proportion of the total effect (% mediated) were projected on to atlas-based cortical effect size maps for CBCL outcomes as annotated below each panel **(c)**. The rule-break scale showed the greatest overall mediation effects (highlighted). All mediation effects were derived from jointly modelling two regressions—the first assessed the ROI using the predictor and the second assessed the CBCL outcome using the ROI as a predictor. Confidence intervals were obtained by bootstrapping 1000 replicates. A threshold of $P < 0.05$ following correction for false discovery was applied to all mediation models in addition to removing effects whose confidence intervals crossed zero. Source data are provided as a source data file.

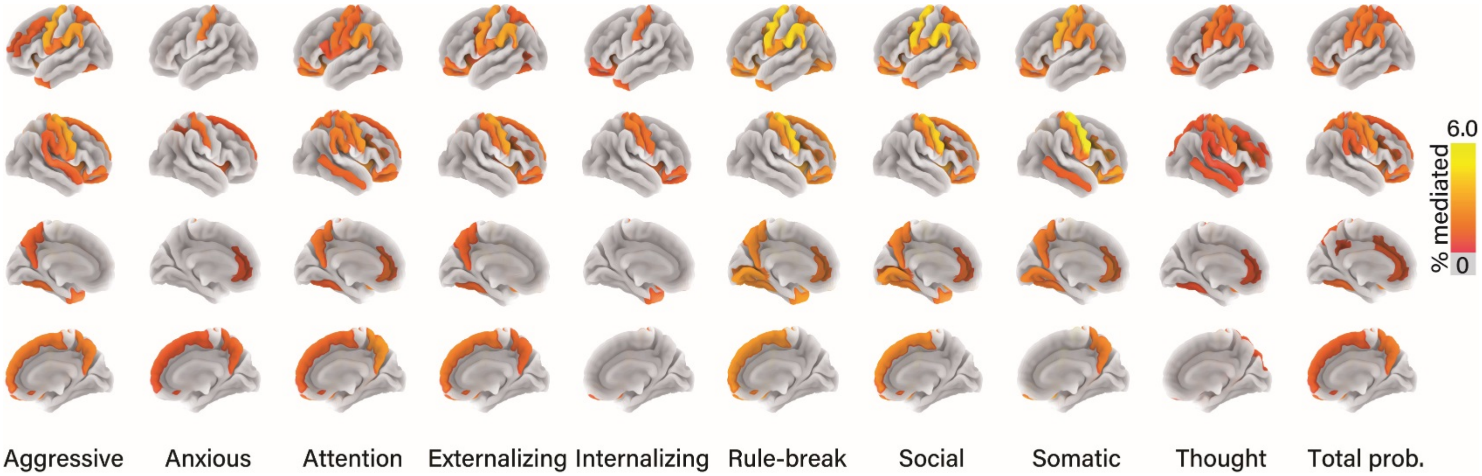

Supplementary Figure 4 Mediation effects for the relationship between the frequency of snoring and abnormal Child Behavior Checklist (CBCL) measures. Covariate-adjusted effect size maps show the regional mediated effects that define the association between the frequency of snoring reported on a five-point scale as 1=never, 2=occasional (once or twice a month), 3=sometimes (once or twice a week), 4=habitual (more than twice a week but not daily), and 5=daily. The CBCL outcomes include the composite and individual scales as annotated below each panel. The outcome variable was a dichotomized variable derived from the cut-off for clinical evaluation with at least a borderline score as shown in Table S1. For social, thought, attention and total problems, arbitrary cut-offs of 65 were used. The mediated effects, expressed as a percentage of the total effect, were calculated from bootstrapped confidence intervals derived from joint modeling of two regressions. The first measures the relationship between the frequency of snoring and the morphometric cortical region of interest (ROI), and the second between the ROI variable and the dichotomized category abnormal CBCL scores defined in Table S1. The mediated effects were projected on four views of atlas-based maps following application of a P value threshold of 0.05, corrected for false discovery. Source data are provided as a source data file.

Supplementary Tables

CBCL category	Mean t-score (± SD)	N of children with borderline t-score (%)	N of children with clinical t-score (%)
Anxious/depressed	53.5 ± 6.0	482 (4.8)	285 (2.8)
Somatic problems	54.9 ± 6.0	536 (5.3)	286 (2.8)
Social problems	52.7 ± 4.7	NA	NA
Thought problems	53.8 ± 5.9	NA	NA
Attention problems	53.8 ± 6.1	NA	NA
Rule-break behavior	52.5 ± 4.8	199 (2.0)	217 (2.1)
Aggressive behavior	52.8 ± 5.4	346 (3.4)	219 (2.2)
Internalizing problems	48.5 ± 10.6	708 (7.0)	986 (9.7)
Externalizing problems	45.6 ± 10.2	432 (4.3)	582 (5.7)
Total problems	45.8 ± 11.2	NA	NA

Supplementary Table 1 Child Behavior Checklist (CBCL) outcomes in the Adolescent Brain and Cognitive Development (ABCD) cohort described by each category subscale. Means and standard deviations (SD) of t-scores are derived by comparing the CBCL raw scores with published age- and gender-based norms. Clinical t-scores are ≥64 for internalizing and externalizing problems, while borderline scores are 60-63 for these scales. For all other scales except those indicated by NA for which cut-offs have not been defined, clinical t-scores are ≥70 and borderline for 65-69. Children meeting the criteria for borderline scores and above are deemed candidates for further evaluation. Source data are provided as a source data file.

oSDB factor score	Age	Sex	Race/ethnicity	Body mass index percentile	Asthma	Income	Education
Breathing problems	0.01	0.04*	0.14**	0.15***	1.80***	0.08	0.01
Gasping	0.01	0.03	0.09***	0.16***	0.62***	0.07**	0.01
Snoring	0.10**	0.01	0.69***	1.14***	0.34***	0.19***	0.12***
Total score	0.05*	0.00	0.70***	1.11***	0.98***	0.23***	0.05*

Supplementary Table 2 Effect size estimates for the relationship between baseline covariates and the parent-reported symptoms of obstructive sleep disordered breathing (oSDB). The frequency of each of the three individual symptoms (nighttime breathing problems, gasping and snoring) and the overall severity was calculated by adding the individual symptom scores. These estimates are determined by measuring the change in the overall proportion of variance (adjusted R^2) in the outcome using a generalized additive model after each of these variables is dropped from the covariate-only model. Asterisks indicate level of statistical significance, * < 0.05, ** between 0.01 and 0.001 and *** < 0.001.

Subcortical structure	Left		Right	
	ΔR^2	P	ΔR^2	P
Thalamus	0.00	0.06	0.00	0.10
Caudate	0.00	0.51	0.00	0.63
Putamen	0.00	0.80	0.00	0.79
Pallidum	0.00	0.48	0.00	0.42
Hippocampus	0.00	0.36	0.00	0.35
Amygdala	0.00	0.29	0.00	0.17
Cerebellum, white matter	0.00	0.70	0.00	0.83
Cerebellum, cortex	0.00	0.17	0.00	0.14

Supplementary Table 3 Effect size estimates for the relationship between obstructive sleep disordered breathing (oSDB) factor score and the volumes of key non-cortical structures. These estimates are determined by measuring the change in the proportion of variance ($\Delta R^2_{adjusted}$ expressed as %) after each of these variables is added to the covariate-only model with scanner serial number as a random effect and testing for statistical significance for the comparison. Results are grouped by the hemisphere of interest (left or right). The P values have not been adjusted for multiplicity.

Supplementary Methods

Baseline demographics

For statistical models, variable conversions were applied in preparation for the additive models used. As the Adolescent Brain Cognitive Development (ABCD) study sampling strategy provided ranges of household income before taxes **(Table 1)**, the median of each income class was first calculated. Due to the positive skew associated with the distribution of income, the natural log of the median was subsequently included in all statistical models. This conversion was performed in accordance with previous large studies of socioeconomic factors and brain structure.[1] Similarly binned data related to the highest parental education was converted to the number of years of education typically associated with the reported educational attainment.

Magnetic Resonance Imaging

Consolidating the previous neuroimaging protocols associated with large studies such as the Pediatric Imaging, Neurocognition, and Genetics (PING) Study (http://www.chd.ucsd.edu/research/ping-study.html)[2] and the Human Connectome Project (https://www.humanconnectome.org)[3], the ABCD study harmonized the collection of high-resolution T1- and T2-weighted images from 21 sites throughout the US. Three 3-Tesla scanner platforms were utilized: Siemens Prisma® (Siemens Medical Solutions USA, Malvern, PA), General Electric Discovery™ MR750 (GE Healthcare, Chicago, IL) and Philips Achieva (Koninklijke Philips, Amsterdam, Netherlands). All images were acquired using standard adult-size multi-channel coils for multiband echo planar imaging. The rationale, parameters and technical details concerning the imaging protocols has been previously described.[4,5]

Use of Generalized Additive Models

In accordance with pre-specified plan for analysis discussed in detail as an online resource accompanying the datasets from the ABCD study (Data Exploration and Analysis Portal, https://deap.nimhda.org), we utilized generalized additive models (GAM). GAM models were chosen for their flexible approach for modelling non-linear covariate effects[6]. These models have been previously employed in studies related to the ABCD cohort[7,8] and other neuroimaging studies[2]. Interpretation of the results from a study of the scale of ABCD is achieved using a combination of effect size estimates[9] and P values. In our study, the contribution of a specific variable to the model was derived by calculating the change in overall variance (adjusted ΔR^2 converted to percent) by assessing the model again after adding the variable to the null model comprised of baseline fixed and random effect covariates only. The gam model from the mgcv (https://people.maths.bris.ac.uk/~sw15190/mgcv) package in R (https://www.r-project.org) was used for this purpose. In the current study, the total household pre-tax income was defined as a smooth term. In addition, the recruitment site and the scanner serial number were included as random effect terms. In assessing interactions, the tensor product interaction of the exposure (obstructive sleep disordered breathing factor score) and household income was included. The unique variance attributed to these interactions were separately calculated by measuring the change in adjusted R^2 from the model without the interaction term.

Mediation Analysis

The key construct of mediation analysis is the determination of the effect attributable to the intermediate variable, whose relationships with the exposure and the outcome variables form the mechanistic basis for the association[10]. This pathway is a conceptual extension of the well-

known and highly cited method described by Baron and Kenny[11]. Although the mediation[12] package used in the current study was initially described for studies in which exposure is randomized to provide the best available estimate of a potential causal association, we utilize the method without explicit characterization of the directional nature of these relationships[13]. The steps for the mediation analysis included two regression equations. First, the morphometric variable associated with a cortical region of interest (ROI) was modeled on the baseline covariates. Next, the CBCL measure was modeled on the ROI variable and baseline covariates. Statistically significant covariates identified for the direct effects of oSDB on CBCL and that on ROI were included in all models. Where the distribution of the outcome was skewed, we used a generalized linear model with a gamma distribution function.[14] The mediation effect was then calculated using the bias-corrected bootstrap approach (n = 1000 replicates)[15]. A detailed discussion of the steps is provided in the technical papers described above. Although a sensitivity analysis is typically recommended for mediation analyses for testing causal hypotheses, the observational nature of the current study assumes that unmeasured confounding could occur from measurement error as both exposure and outcome were semi-quantitatively estimated from parental reports. Furthermore, the multivariate models iteratively analyzed each of the 148 cortical ROIs separately (74 for each hemisphere) and therefore all P values were adjusted for false discovery using the Benjamini-Hochberg method[16].

Supplementary References

1 Noble KG, Houston SM, Brito NH, *et al.* Family income, parental education and brain structure in children and adolescents. *Nat Neurosci* 2015; **18**: 773–8.

2 Jernigan TL, Brown TT, Hagler DJ, *et al.* The Pediatric Imaging, Neurocognition, and Genetics (PING) Data Repository. *Neuroimage* 2016; **124**: 1149–54.

3 Van Essen DC, Ugurbil K, Auerbach E, *et al.* The Human Connectome Project: A data acquisition perspective. *Neuroimage* 2012; **62**: 2222–31.

4 Casey BJ, Cannonier T, Conley MI, *et al.* The Adolescent Brain Cognitive Development (ABCD) study: Imaging acquisition across 21 sites. *Dev Cogn Neurosci* 2018; **32**: 43–54.

5 Hagler DJ, Hatton S, Cornejo MD, *et al.* Image processing and analysis methods for the Adolescent Brain Cognitive Development Study. *Neuroimage* 2019; **202**: 116091.

6 Hastie T, Tibshirani R. Generalized Additive Models. *Statist Sci* 1986; **1**: 297–310.

7 Dick AS, Garcia NL, Pruden SM, *et al.* No evidence for a bilingual executive function advantage in the nationally representative ABCD study. *Nat Hum Behav* 2019; **3**: 692–701.

8 Gonzalez MR, Palmer CE, Uban KA, Jernigan TL, Thompson WK, Sowell ER. Economic, social, and physiological resilience predict brain structure and cognitive performance in 9 - 10-year-old children. *bioRxiv* 2019; : 852988.

9 Nakagawa S, Cuthill IC. Effect size, confidence interval and statistical significance: a practical guide for biologists. *Biol Rev Camb Philos Soc* 2007; **82**: 591–605.

10 Imai K, Keele L, Tingley D. A general approach to causal mediation analysis. *Psychol Methods* 2010; **15**: 309–34.

11 Baron RM, Kenny DA. The moderator-mediator variable distinction in social psychological research: conceptual, strategic, and statistical considerations. *J Pers Soc Psychol* 1986; **51**: 1173–82.

12 Tingley D, Yamamoto T, Hirose K, Keele L, Imai K. mediation: R Package for Causal Mediation Analysis. *Journal of Statistical Software* 2014; **59**: 1–38.

13 Imai K, Keele L, Tingley D, Yamamoto T. Unpacking the black box of causality: Learning about causal mechanisms from experimental and observational studies. *American Political Science Review* 2011; **105**: 765–789.

14 Ng VKY, Cribbie RA. Using the gamma generalized linear model for modeling continuous, skewed and heteroscedastic outcomes in psychology. *Current Psychology: A Journal for Diverse Perspectives on Diverse Psychological Issues* 2017; **36**: 225–35.

15 DiCiccio TJ, Efron B. Bootstrap Confidence Intervals. *Statistical Science* 1996; **11**: 189–212.

16 Benjamini Y, Hochberg Y. Controlling the False Discovery Rate: A Practical and Powerful Approach to Multiple Testing. *Journal of the Royal Statistical Society: Series B (Methodological)* 1995; **57**: 289–300.
