# Supplementary material for: Associations between frontal lobe structure, parent-reported obstructive sleep disordered breathing and childhood behavior in the ABCD dataset
Source: Nat Commun. 2021 Apr 13;12:2205. doi: 10.1038/s41467-021-22534-0 (PMC8044120; doi:10.1038/s41467-021-22534-0)
Supplement: Supplementary file 3 — Reporting Summary [file 41467_2021_22534_MOESM3_ESM.pdf]

nature research

Corresponding author(s): Amal Isaiah

Last updated by author(s): 2/08/2021

Reporting Summary

Nature Research wishes to improve the reproducibility of the work that we publish. This form provides structure for consistency and transparency in reporting. For further information on Nature Research policies, see our Editorial Policies and the Editorial Policy Checklist.

Statistics

For all statistical analyses, confirm that the following items are present in the figure legend, table legend, main text, or Methods section.

n/a	Confirmed	
[ ]	[x]	The exact sample size (*n*) for each experimental group/condition, given as a discrete number and unit of measurement
[ ]	[x]	A statement on whether measurements were taken from distinct samples or whether the same sample was measured repeatedly
[ ]	[x]	The statistical test(s) used AND whether they are one- or two-sided<br>*Only common tests should be described solely by name; describe more complex techniques in the Methods section.*
[ ]	[x]	A description of all covariates tested
[ ]	[x]	A description of any assumptions or corrections, such as tests of normality and adjustment for multiple comparisons
[ ]	[x]	A full description of the statistical parameters including central tendency (e.g. means) or other basic estimates (e.g. regression coefficient) AND variation (e.g. standard deviation) or associated estimates of uncertainty (e.g. confidence intervals)
[ ]	[x]	For null hypothesis testing, the test statistic (e.g. *F*, *t*, *r*) with confidence intervals, effect sizes, degrees of freedom and *P* value noted<br>*Give P values as exact values whenever suitable.*
[x]	[ ]	For Bayesian analysis, information on the choice of priors and Markov chain Monte Carlo settings
[x]	[ ]	For hierarchical and complex designs, identification of the appropriate level for tests and full reporting of outcomes
[ ]	[x]	Estimates of effect sizes (e.g. Cohen's *d*, Pearson's *r*), indicating how they were calculated

Our web collection on statistics for biologists contains articles on many of the points above.

Software and code

Policy information about availability of computer code

Data collection	Commercially available Redcap software (v9.1) was used for the harmonized collection of demographic data from all 21 sites. Freesurfer (v5.3.0) was used for centralized processing of MRI images as part of Multi-Modal Processing Stream (MMPS), a software package developed and maintained in-house at the Center for Multimodal Imaging and Genetics (CMIG) at the University of California, San Diego (UCSD). Additional details are provided by https://abcdstudy.org.
Data analysis	R (v3.6.1), R package mgcv (v1.8) for generalized additive models, nilearn package (v0.5.2) for generation of atlas-based maps. All code available for sharing upon request.

For manuscripts utilizing custom algorithms or software that are central to the research but not yet described in published literature, software must be made available to editors and reviewers. We strongly encourage code deposition in a community repository (e.g. GitHub). See the Nature Research guidelines for submitting code & software for further information.

Data

Policy information about availability of data

All manuscripts must include a data availability statement. This statement should provide the following information, where applicable:

- Accession codes, unique identifiers, or web links for publicly available datasets
- A list of figures that have associated raw data
- A description of any restrictions on data availability

The ABCD study follows open science policies in line with the NIH policy on rigor and reproducibility. The dataset obtained from the ABCD study (https://nda.nih.gov/abcd) following a data use agreement. Data used in the preparation of this article were obtained from the Adolescent Brain Cognitive Development (ABCD) Study (https://abcdstudy.org), held in the National Institutes of Mental Health (NIMH) Data Archive (NDA). A digital object identifier is available (https://

doi.org/10.15154/1520518) for the subset of data used in the current study. Custom R code for the generation of figures in the manuscript are hosted at https://github.com/sjoh2574/oSDB.git.

Field-specific reporting

Please select the one below that is the best fit for your research. If you are not sure, read the appropriate sections before making your selection.

☐ Life sciences ☒ Behavioural & social sciences ☐ Ecological, evolutionary & environmental sciences

For a reference copy of the document with all sections, see nature.com/documents/nr-reporting-summary-flat.pdf

Behavioural & social sciences study design

All studies must disclose on these points even when the disclosure is negative.

Study description	This is a cross-sectional analysis of qualitative and quantitative outcomes from the ABCD study (https://abcdstudy.org). Children aged 9-10 years were recruited from the community from 21 sites across the United States are being followed for the next decade until adulthood. We used the baseline dataset (v.2.0.1) comprising 11,875 children and included 10,140 children in whom complete observations were available for demographic, cognitive, sleep questionnaires and structural MRI scans.
Research sample	The ABCD dataset follows the open-science format of the original study, which houses the data at the National Institutes of Mental Health Data Archive (NDA, https://nda.nih.gov/abcd). The study population included 9-10-year-old 'typically-developing' children recruited from 21 institutions throughout the United States (ABCD Consortium). The full list of locations is provided by https://abcdstudy.org/study-sites. While the study sample was conceived to represent the US population in its socioeconomic and racial diversity, it does not make claims concerning representation of all aspects of preadolescents in the United States (https://jamanetwork.com/journals/jamapediatrics/article-abstract/2737910). The sample size ensures adequate power for the detection of small to medium effects for the various exploratory analyses over theduration of the study and accounts for ~10% attrition over the course of the study (Garavan et al. 2018).The baseline dataset used in the study was released in October 2019 as v.2.0.1 following an issued fix for scanner-related incongruencies.
Sampling strategy	Baseline variables include demographic and anthropometric characteristics, along with cognitive and subjective sleep assessments. By its design, the ABCD study approximates the diversity of the U.S. population on sex, race and ethnicity, and socioeconomic status. The recruitment strategy replicates a multi-stage probability sample of eligible children derived from a national distribution of the eligible sites, the schools within the vicinity of each site and the children recruited from these schools (Garavan et al. 2018).
Data collection	The sleep disturbance scale for children (SDSC) is a high-quality instrument for the detection of sleep-related abnormalities. The questionnaire was filled by the primary caregiver based on their observations over the preceding six months. The sleep related breathing disorder factor score was derived by adding the individual symptoms scored on a Likert-type scale. The symptom survey replicates previous large scale parental surveys performed in person or in mail (Li et al. 2010; Li et al. 2012). Behavior in the ABCD cohort was assessed using the Child Behavior Checklist (CBCL), a validated and widely-used assessment of childhood behavior spanning emotional, social and psychological domains. The test, administered in the ABCD study to the primary caregiver, has excellent psychometric properties and provides additional syndrome scores in the following categories: anxious/depressed, somatic complaints, social problems, thought problems, attention problems, rule-breaking behavior and aggressive behavior, in addition to three composite scores: internalizing, externalizing and total problems. Raw scores were converted to t-scores using gender- and age-based norms from population-based studies, with higher scores indicating more severe behavioral problems. All children underwent structural brain MRI according to standardized protocols (full details below). Locally acquired T1-weighted images were processed at the Data Analysis Informatics and Resource Center (DAIRC) of the ABCD study. The caregiver was in the immediate vicinity for all in-person assessments. Scans were performed in the presence of the study team (MRI technician) without the parent present in the room in most instances. Due to the observational nature of the study, the study team was not blinded to the experimental condition(s) or the hypotheses associated with the study.
Timing	Baseline dataset covered children recruited between September 2016 and October 2018; the data collection is ongoing.
Data exclusions	Although data was available for 11,875 children from the v2.0.1 release, 10,140 were finally included due to incomplete or missing observations.
Non-participation	None of the participants dropped out of the 10,140 children included from the baseline dataset.
Randomization	Children were not randomized in this observational study as there were no experimental groups. Covariate control was achieved using the statistical models used that incorporated age, sex, income, educational status, body mass index and the presence of asthma.

Reporting for specific materials, systems and methods

We require information from authors about some types of materials, experimental systems and methods used in many studies. Here, indicate whether each material, system or method listed is relevant to your study. If you are not sure if a list item applies to your research, read the appropriate section before selecting a response.

Materials & experimental systems

n/a	Involved in the study
☒	☐ Antibodies
☒	☐ Eukaryotic cell lines
☒	☐ Palaeontology and archaeology
☒	☐ Animals and other organisms
☐	☒ Human research participants
☒	☐ Clinical data
☒	☐ Dual use research of concern

Methods

n/a	Involved in the study
☒	☐ ChIP-seq
☒	☐ Flow cytometry
☐	☒ MRI-based neuroimaging

Human research participants

Policy information about studies involving human research participants

Population characteristics

See above.

Recruitment

The current dataset (v2.0.1) includes 11,875 children enrolled between September 1, 2016 and November 1, 2018. Baseline variables include demographic and anthropometric characteristics, along with cognitive and subjective sleep assessments. By its design, the ABCD study approximates the diversity of the U.S. population on sex, race and ethnicity, and socioeconomic status. The recruitment strategy replicates a multi-stage probability sample of eligible children derived from a national distribution of the eligible sites, the schools within the vicinity of each site and the children recruited from these schools, and included -- but were not limited to -- the use of electronic and paper-based materials, mailing lists, camp-based and directly approaching families. This strategy resembles the American Community Survey, however, some selection bias is possible owing to the location of the recruitment sites and imaging centers within urban areas. Substantial attention was paid to ensure adequate representation of racial and socioeconomic subgroups although there is an over-representation of higher-income families. The ABCD continues to monitor recruitment and continued assessments to ensure that deviation from the original sample constitution is minimized. In the current dataset, there appears to be some over-representation of higher income families. Full details of recruitment and retention strategies are provided by Garavan et al. (2018).

Ethics oversight

The institutional review boards (IRB) of the following institutions approved the study, in addition to the central IRB at University of California, San Diego: University of Maryland, Baltimore, University of Colorado Boulder, University of Minnesota, the Laureate Institute for Brain Research, Oregon Health and Science University, University of Vermont, University of Pittsburgh, Virginia Commonwealth University, University of Rochester, University of Florida, the Medical University of South Carolina, University of Michigan, University of Minnesota, University of Utah, SRI International, University of Wisconsin-Milwaukee, Children's Hospital of Los Angeles, Florida International University, Washington University in St. Louis, and Yale University.

Note that full information on the approval of the study protocol must also be provided in the manuscript.

Magnetic resonance imaging

Experimental design

Design type

Only structural MRI data is included in the current study although task-related MRI was collected during the study.

Design specifications

A full list of neuroimaging parameters are provided by https://abcdstudy.org/images/ Protocol_Imaging_Sequences.pdf.

Behavioral performance measures

Not collected for structural imaging.

Acquisition

Imaging type(s)

Structural

Field strength

3.0T

Sequence & imaging parameters

The ABCD imaging protocol is harmonized across three 3T scanner platforms (Siemens Prisma, General Electric (GE) 750 and Philips) and uses multi-channel coils capable of multiband echo planar imaging (EPI) acquisitions, using a standard adult-size coil. The three scanners used the following settings. Echo-planar, T1 256 x 256 matrix size, 225 slices, 256 x 240 FOV, 1 mm resolution in all axes, TR = 6.31 ms; TE = 2.9 ms and TI = 1060 ms; flip angle = 8 deg; 1.5 x 2.2 parallel imaging; 1.5 x 2.2 mm parallel imaging; multiband off. Full details are provided in Casey et al. (2018).

Area of acquisition

Whole brain morphometry including thickness, area and volume.

Diffusion MRI ☐ Used ☒ Not used

Preprocessing

Preprocessing software

Excerpt from Hagler et al. (2019): "...Multi-Modal Processing Stream (MMPS), a software package developed and maintained

Preprocessing software	in-house at the Center for Multimodal Imaging and Genetics (CMIG) at the University of California, San Diego (UCSD). MMPS provides large- scale, standardized processing and analysis of multimodality neuroimaging data on Linux workstations and compute clusters. MMPS is a toolbox of primarily MATLAB functions, but also includes python, sh, csh scripts, and C++ compiled executables. MMPS also relies upon a number of publicly available neuroimaging software packages, including FreeSurfer (Fischl, 2012), Analysis of Functional NeuroImages (AFNI) (Cox, 1996), and FMRIB Software Library (FSL) (Jenkinson et al., 2012; Smith et al., 2004)..."
Normalization	Detailed steps provided by Hagler et al. (2019) and Casey et al. (2018). Excerpts provided below:<br><br>"...A standard scan session includes sMRI series (T1w and T2w), one dMRI series, four rs- fMRI series, and three sets of two task-fMRI series (MID, SST, and EN-back). Minimal details of the imaging protocol are provided here to contextualize the following description of the processing pipeline; additional details have been published previously (Casey et al., 2018). Scan sessions typically require ~2 hours to complete, including a mid-session rest break if the child requests one; they are sometimes split into two separate sessions that take place within one week of each other (3.4% of participants included in ABCD Data Release 1.1). Over 78% of the participants included in ABCD Data Release 1.1 successfully completed the entire image acquisition protocol..."<br><br>Further, "...T1w and T2w structural images are corrected for gradient nonlinearity distortions using scanner-specific, nonlinear transformations provided by MRI scanner manufacturers (Jovicich et al., 2006). T2w images are registered to T1w images using mutual information (Wells et al., 1996) after coarse, rigid-body pre-alignment via within-modality registration to atlas brains. MR images are typically degraded by a smooth, spatially varying artifact (receive coil bias) that results in inconsistent intensity variations. Intensity inhomogeneity correction is performed by applying smoothly varying, estimated B1-bias fields, using a novel implementation that is similar in purpose to commonly used bias field correction methods (Ashburner and Friston, 2000; Sled et al., 1998). Specifically, B1-bias fields are estimated using sparse spatial smoothing and white matter segmentation, with the assumption of uniform T1w (or T2w) intensity values within white matter. To normalize T1w and T2w intensities across participants, a target white matter intensity value of 110 is used so that after bias correction, white matter voxel intensities are centered on that target value and all other voxels are scaled relatively. The value of 110 was chosen to match the white matter value assigned by the standard bias correction used by FreeSurfer. The white matter mask, defined using a fast, atlas-based, brain segmentation algorithm, is refined based on a neighborhood filter, in which outliers in intensity -- relative to their neighbors within the mask -- are excluded from the mask. A regularized linear inverse, implemented with an efficient sparse solver, is used to estimate the smoothly varying bias field. The stiffness of the smoothing constraint was optimized to be loose enough to accommodate the extreme variation in intensity that occurs due to proximity to the imaging coils, without overfitting local intensity variations in white matter. The bias field is estimated within a smoothed brain mask that is linearly interpolated to the edge of the volume in both directions along the inferior-superior axis, avoiding discontinuities in intensity between brain and neck..."<br><br>"...Images are rigidly registered and resampled into alignment with a custom, in-house atlas brain that has 1.0 mm isotropic voxels and is roughly aligned with the anterior commissure / posterior commissure (AC/PC) axis, facilitating standardized viewing and analysis of brain structure. For most participants, a single scan of each type is collected. If multiple scans of a given type are obtained, only one is used for processing and analysis. Results of manual quality control (QC) performed prior to the full image processing are used to exclude poor quality structural scans (refer to the Quality Control section). If there is more than one acceptable scan of a given type, the scan with the fewest issues noted is used. In case of a tie, the final acceptable scan of the session is used..."
Normalization template	As above.
Noise and artifact removal	Anatomical 3D T1- and 3D T2-weighted images were collected using prospective motion correction (PROMO) on the GE (White et al., 2010), Volumetric Navigators (vNav) for prospective motion correction and selective reacquisition on the Siemens and when available on the Philips platform (Tisdall et al., 2012).
Volume censoring	As above.

Statistical modeling & inference

Model type and settings	Generalized additive models used to incorporate flexible non-linear modeling of variables such as household income. BRIEF outcomes were converted to t-scores. Null-effects model (covariates only as fixed effects with study site as random effect) compared with covariates + independent variable under consideration (e.g. SDSC symptom and total scores) and change in R2, P values calculated based on a likelihood ratio test. For imaging data, mass univariate modeling was performed using generalized additive models including scanner serial number and study sites as covariates. Model fit and effects were calculated by change in R2 at each ROI location determined following an FDR threshold of 0.05. For mediation analyses, joint modeling of two regressions performed with calculation of effect size as above and identical FDR threshold. n = 1000 iterations for bootstrapped estimates of confidence intervals.
Effect(s) tested	Direct and indirect using mediation of mixed effect models. Change in R2 used as primary effect size, FDR correction applied as above.

Specify type of analysis: ☐ Whole brain ☐ ROI-based ☒ Both

Anatomical location(s)	Probabilistic atlas (Destrieux) and global whole brain measures were used.
Statistic type for inference (See Eklund et al. 2016)	Change in R2 and P value for likelihood test of two models: covariate-only model and covariates + independent variables.

Correction	FDR using B-H method.

Models & analysis

n/a	Involved in the study
☒	☐ Functional and/or effective connectivity
☒	☐ Graph analysis
☒	☐ Multivariate modeling or predictive analysis
